# Postharvest preservation efficacy and optimization strategies of fresh cut flowers: a meta-analysis and machine learning approach

**DOI:** 10.1093/hr/uhaf227

**Published:** 2025-09-03

**Authors:** Yuyang Wu, Jun Zhu, Jingjing Zhang, Yu Zhang, Jiahui Tang, Jinqi Miao, Yue Sun, Jinhua Zou

**Affiliations:** Tianjin Key Laboratory of Animal and Plant Resistance, College of Life Sciences, Tianjin Normal University, Tianjin, China; Tianjin Key Laboratory of Animal and Plant Resistance, College of Life Sciences, Tianjin Normal University, Tianjin, China; Tianjin Key Laboratory of Animal and Plant Resistance, College of Life Sciences, Tianjin Normal University, Tianjin, China; Tianjin Key Laboratory of Animal and Plant Resistance, College of Life Sciences, Tianjin Normal University, Tianjin, China; Tianjin Key Laboratory of Animal and Plant Resistance, College of Life Sciences, Tianjin Normal University, Tianjin, China; Tianjin Key Laboratory of Animal and Plant Resistance, College of Life Sciences, Tianjin Normal University, Tianjin, China; State Key Laboratory of Livestock and Poultry Biotechnology Breeding, College of Biological Sciences, China Agricultural University, Beijing, China; Tianjin Key Laboratory of Animal and Plant Resistance, College of Life Sciences, Tianjin Normal University, Tianjin, China

## Abstract

Cut flowers are favored globally for their ornamental value, but their commercial value is limited by their short vase life, which depends closely on the postharvest preservation technology of cut flowers. Currently, complex types of preservatives and a variety of preservation methods have been used, but there is a lack of summary and comparison of them. In this study, 45 publications were synthesized and analyzed through meta-analysis and machine learning. The meta-analysis results showed that: (i) pulse treatments demonstrated superior vase life extension over conventional vase solution treatments by acutely enhancing antioxidant enzyme activity and suppressing ethylene biosynthesis, but their transient nature necessitated subsequent vase solution treatment maintenance for optimal floral appearance. (ii) As unique preservatives, nanomaterials had advantages in water balance and antimicrobial protection, which required synergistic integration with other preservatives to further enhance antioxidant capacity and supply nutrient. (iii) Plant species specificity needed to be taken into account when choosing the preservative types for vase solution treatment. The model prediction results of machine learning revealed that identical preservatives exhibited distinct differences when applied as pulse treatments versus vase solution treatments, indicating pulse treatment could amplify the preservation effect of preservatives. Based on the above results, an optimized implementation protocol was proposed: initial pulse treatment with nanomaterials, then species-specific preservatives addressed as supplement vase solutions treatment. Our verification experiments further validated that the optimized preservation protocol was effective in cut roses (*Rosa hybrida* L. cv. Carola). The findings provided mechanistic guidance for optimizing preservative combinations, and a theoretical foundation and direction for future research.

## Introduction

Cut flowers, one of the most popular ornamental plants in the world, have a short display time [1]. Extending the vase life of cut flowers and delaying their wilting have long been research topics in horticulture. The vase life is primarily influenced by flowers’ genotype, preharvest, and postharvest conditions [2]. Among these factors, postharvest conditions directly impact the commercial value of cut flowers and the economic outcomes of the floriculture industry [3]. Furthermore, postharvest treatments are the most straightforward and effective approach to prolonging the vase life of cut flowers, making them become a widely studied topic [4]. Once separated from the parent plants, flowers continue to undergo complex metabolic processes, including water imbalance, reactive oxygen species (ROS) accumulation, and nutrient depletion, etc. [4]. Various postharvest strategies have been explored to extend the postharvest life of cut flowers, including the application of preservatives, regulation of the color of the irradiated light, and using acidic water at pH 5.5 [5, 6]. Among these approaches, the use of chemical preservatives has received the most extensive research attention [4].

Research on postharvest preservation agents for cut flowers has advanced rapidly, with diverse substances showing distinct effects on vase life. Salicylic acid (SA) is a simple phenolic compound, and current reports suggest that SA retards ion leakage from petals, increases antioxidant enzyme activity, and reduces fresh weight (FW) loss and membrane lipid peroxidation [7]. Plant growth regulators, including cytokinins (CTK), gibberellins (GA_3_), and abscisic acid (ABA), have also been studied for cut flower preservation [8–10]. Recent studies have applied nanomaterials in preservation research due to their unique physicochemical properties. Graphene oxide, for instance, has shown great potential due to its high surface area and adsorption capacity. These properties help lower bacterial infection risks, reduce water transport barriers from xylem blockage, and then maintain water balance, thereby extending vase life [11, 12].

Current efforts focus on developing composite preservatives combining multiple substances for synergistic effects. For example, combining nanomaterials with plant hormones significantly improves ornamental vase life [13], while antimicrobial–sucrose mixtures alleviate water stress and oxidative damage [14]. However, most combinations remain empirical, lacking mechanistic understanding of the interactions between components. In addition, there is still a lack of comparative studies on the preservative efficacy of these agents. Therefore, it is necessary to systematically summarize the existing research, analyze the preservative mechanisms of different agents, and optimize storage strategies to achieve optimal preservation results [15].

Meta-analysis has been widely used in agricultural research to statistically integrate data from similar studies. Based on the experimental data of existing articles, meta-analysis surpasses conventional approaches, through exploring the relationship between the measured physiological parameters and the influencing factors to reveal the laws and connections between the existing dataset and find the problems in the current research [16, 17]. Machine learning, as a new analytical methodology, is useful in medical and environmental research and can be applied to the analysis of research in the field of fresh cut flower preservation [18, 19]. By machine learning, the experimental data from published articles are integrated, multiple models are trained, and the best fitting models are filtered out. Then their SHAP (SHapley Additive exPlanations‌) values are calculated to quantify feature contributions [20], and the best fitting model can be used to predict the missing values.

## Results

### Effect of preservative treatment methods, types and concentration, and plant species on the preservation efficacy of cut flowers

**Figure 1:**
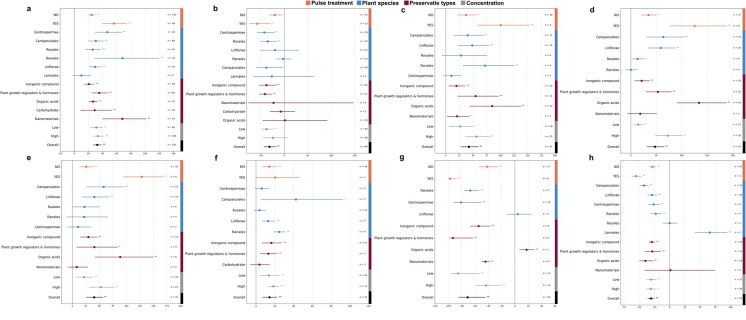
Forest plot showing the effect sizes and 95% CI for eight physiological parameters across the four categories of influencing factors. The *x*-axis represents the relative change (percent) in each parameter compared to that in control group, calculated using the response ratio (RR). The length of each horizontal line represents the calculated effect size of each subgroup, with shorter intervals indicating greater precision. The vertical line at 0% denotes no effect. There is no significant effect when the horizontal line intersects with the vertical line, a negative effect when the horizontal line is on the left of the vertical line, and a positive effect when the horizontal line is on the right of the vertical line. *P < 0.05, and **P < 0.01. Sample sizes (*n*) are shown at the right of each horizontal line. (a) vase life, (b) RFW, (c) SOD (superoxide dismutase), (d) CAT (catalase), (e) POD (peroxidase), (f) floral diameter, (g) ethylene content, and (h) MDA (malondialdehyde).

Figure 1 shows the effects of four factors (including whether pulse treatment, plant species, preservative types, and preservative concentration) on eight measured physiological parameters of preservation (including vase life, relative fresh water, SOD (superoxide dismutase), CAT (catalase), POD (peroxidase), floral diameter, ethylene content, MDA (malondialdehyde)). Figure 1a demonstrated that preservative treatments significantly extended cut flower vase life (*P* < 0.01), except in Lamiales species. As for vase life, pulse treatment, Ranales species, and nanomaterials showed superior preservation efficacy. Notably, preservative concentration variations did not produce significant differences based on the existing articles. Pulse treatment, plant growth regulators and hormones, inorganic compound preservatives, and low concentration preservatives all significantly moderated FW loss (*P* < 0.05) (Fig. 1b). Preservative treatments enhanced the activities of three antioxidant enzymes (SOD, CAT, and POD), showing significant overall increases (*P* < 0.01) (Figs 1c–e). It was notable that nanomaterials only significantly elevated SOD activity (*P* < 0.05), while other antioxidant enzymes (CAT, POD) showed no marked changes. Pulse treatment outperformed vase solution treatment in boosting antioxidant enzyme activities, and higher preservative concentrations demonstrated stronger enhancement effects on antioxidant enzyme activities than lower concentrations. Carbohydrate preservatives had no significant effect on flower diameter expansion (Fig. 1f). Ethylene content reduction occurred in most treatments (*P* < 0.01), except in Liliflorae species and organic acid treatment (Fig. 1g). Pulse treatment effectively reduced MDA levels compared to vase solution treatment (Fig. 1h), though the effects on Rosales were nonsignificant. Nanomaterials did not show significant impact on MDA content. The observation showed increased MDA content in Lamiales (*n* = 1), while the number of included studies was small, and this result was only indicative.

### Correlation and linear relationship between the measured parameters

**Figure 2:**
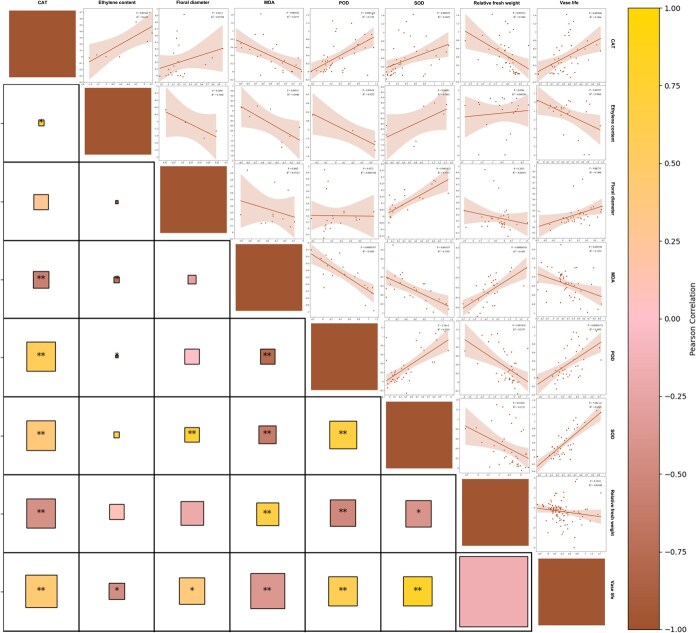
Heat map of Pearson’s correlation and linear relationship plot between eight physiological parameters’ lnRR. The size of the box indicates the number of experiments included in the analysis. The closer the Pearson correlation coefficient is to 1, the stronger the positive correlation; the closer it is to -1, the stronger the negative correlation. *P* < 0.05 indicates statistical significance. *P < 0.05, **P < 0.01. R^2^ indicates the fit goodness in the results of the linear fit.

Figure 2 revealed that preservative-induced changes in vase life showed significant correlations (*P* < 0.05) with all measured parameters except relative FW variations. Vase life changes negatively correlated with ethylene content variations and MDA content changes. Relative FW variations exhibited negative correlations with the changes of SOD, CAT, and POD activities (*P* < 0.05), but positive correlations with MDA accumulation (*P* < 0.01). Strong positive linear relationships emerged among the changes in SOD, POD, and CAT activities (*P* < 0.01), while all three antioxidant enzymes showed negative linear correlations with MDA content variations (*P* < 0.01). Notably, SOD activity changes demonstrated a positive linear relationship with floral diameter variations (Fig. 2).

**Figure 3:**
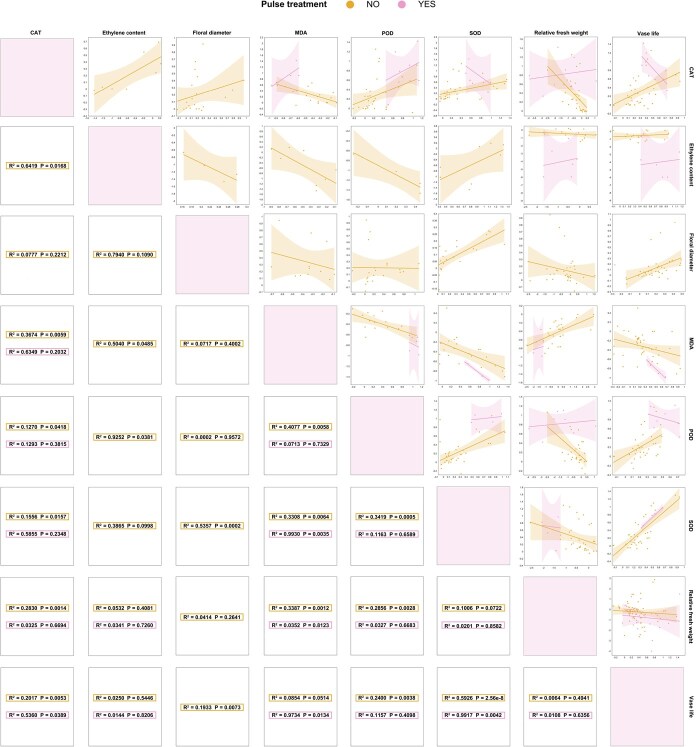
Linear relationship plot between the eight parameters’ lnRR. *P* < 0.05 indicates statistical significance. R^2^ indicates the fit goodness in the results of the linear fit.

Pulse treatment induced distinct correlation patterns compared to vase solution treatment (Fig. 3). For instance, between pulse treatment and no pulse treatment, there were divergences in the correlations in the changes of CAT and POD activities and changes of other measured parameters. In pulse-treated flowers, CAT activity variations were negatively correlated with SOD level changes, and CAT activity variations were positively correlated with MDA content changes. Both SOD and POD activity changes were positively correlated with relative FW, but negatively with vase life.

Figures S1 and S2 showed that, regarding the types of cut flowers or preservatives, there was generally no divergence in the relationships between the changes in different parameters. General analysis showed no significant correlation between vase life changes and relative FW variations, but there were significant positive correlations (*P* < 0.05) in the changes of these parameters, referring to the plant species of Campanulales and Centrospermae, and the preservative types of nanomaterials and organic acid (Figs S1 and S2). Notably, Campanulales uniquely exhibited a significant negative linear correlation between floral diameter changes and relative FW variations (*P* < 0.01), which was different from other plant species (Fig. S1).

### SHAP values explaining the contribution of influencing factors

**Figure 4:**
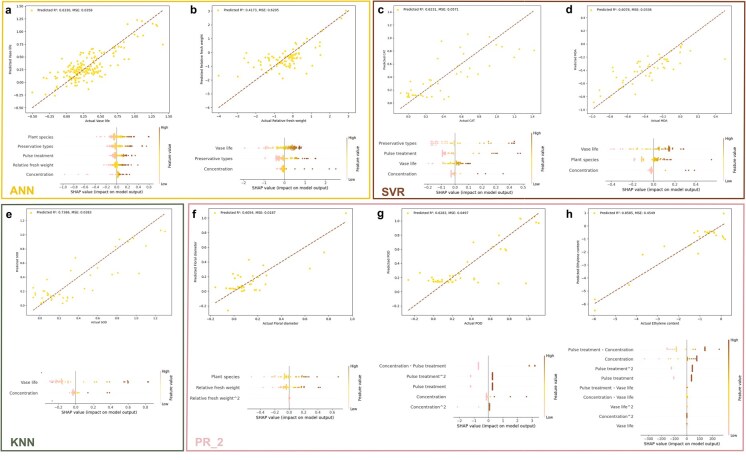
Best performing predictive models for eight parameter lnRR constructed based on machine learning and their SHAP values. (a) vase life, (b) RFW, (c) CAT, (d) MDA, (e) SOD, (f) floral diameter, (g) POD, and (h) ethylene content. (a) and (b) used ANN (Artificial Neural Network). (c) and (d) used SVR (Support Vector Regression). (e) used KNN (K-Nearest Neighbors). (f), (g), and (h) used quadratic polynomial regression (PR_2). R^2^ indicates the goodness of fit. MSE, mean squared error.

Figure 4 displays the best performing models for ln response ratio (lnRR) of different influencing factors and their corresponding SHAP values. The values of the best coefficient of determination (*R*^2^) were 0.8585 in ethylene content model (Fig. 4h), 0.7386 in SOD model (Fig. 4e), 0.6330 in vase life model (Fig. 4a), 0.6283 in POD model (Fig. 4g), 0.6231 in CAT model (Fig. 4c), 0,6078 in MDA model (Fig. 4d), and 0.6054 in floral diameter model (Fig. 4f). Through employing advanced modeling strategies, the *R*^2^ values for all models were higher than 0.6 except the model of relative FW in Fig. 4b, which were acceptable, compared to the accuracy of the predictive models of related studies [21, 22].

SHAP analysis revealed the contribution of influencing factors across different models. In the vase life model, plant species emerged as the primary contributor, followed by preservative types, pulse treatment, relative FW, and preservative concentration (Fig. 4a). In the models of relative FW, MDA, and SOD, vase life showed high contributions (Fig. 4b, d, e). Preservative types exhibited dominant influence in the CAT model (Fig. 4c). When employing polynomial regression for model construction, SHAP values revealed higher order term contributions. Plant species and relative FW were key predictors in the floral diameter and MDA models (Fig. 4d and f). Pulse treatment and preservative concentration dominated feature importance (Fig. 4g and h) in the POD and ethylene models.

### Model predictive analysis

**Figure 5:**
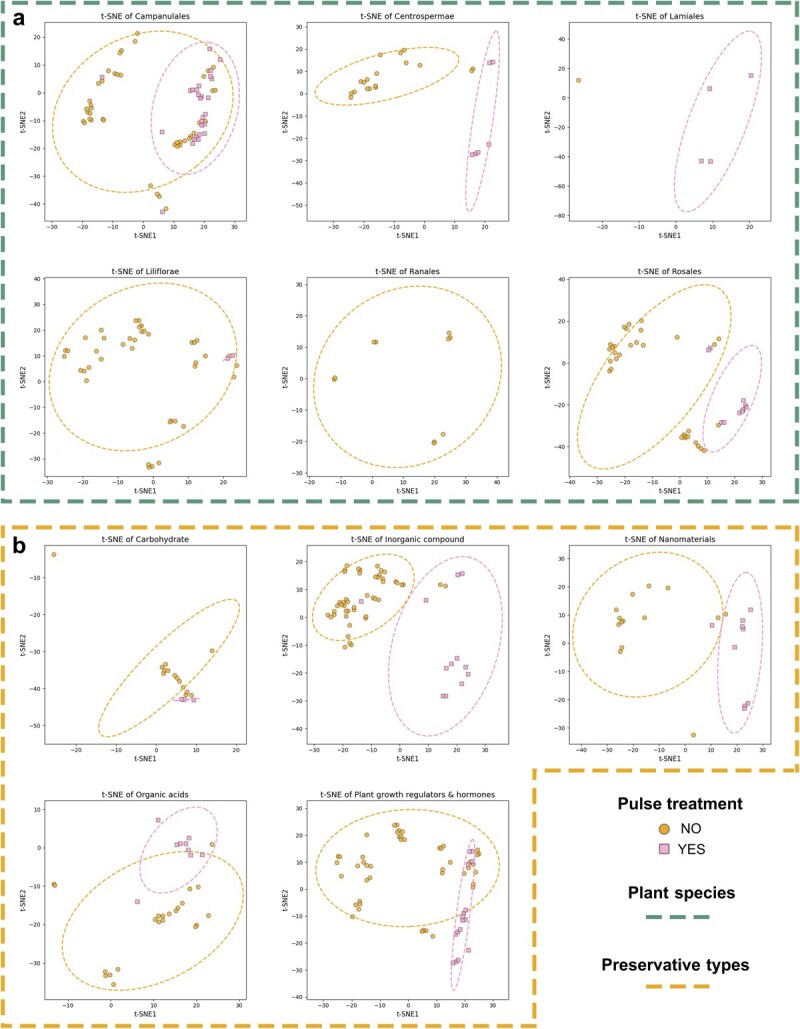
Analysis of t-SNE after the prediction of missing values using the best performing models. (a) Based on species types. (b) Based on preservative types. Ellipses are constructed based on a 95% confidence level.

Despite limitation in the prediction capability, the missing values were predicted using the aforementioned best performing models, and the completed dataset was ultimately performed to t-distributed stochastic neighbor embedding (t-SNE) analysis (Fig. 5). t-SNE analysis is a nonlinear dimensionality reduction method for visualization of high-dimensional data, and is particularly good at capturing local structure in the data, helping to map high-dimensional data into 2D or 3D space for easy visual analysis. It was found that pulse treatment had a significant effect on the preservation of cut flowers, so pulse treatment was used as the basis for separation; plant species (Fig. 5a) and preservative types (Fig. 5b) were used as labels to observe the separation results. As for plant species, it was worth noting that Campanulales showed minimal sensitivity to pulse treatment, contrasting with marked responses in other plant species (Fig. 5a). Regardless of applying different preservative types, such as inorganic compounds, plant growth regulators and hormones, and nanomaterials, divergency occurred between pulse treatment and vase solution treatment (Fig. 5b). In general, a clear separation between pulse treatment and vase solution treatment was found (Fig. 5).

### Verification experiment results

**Figure 6:**
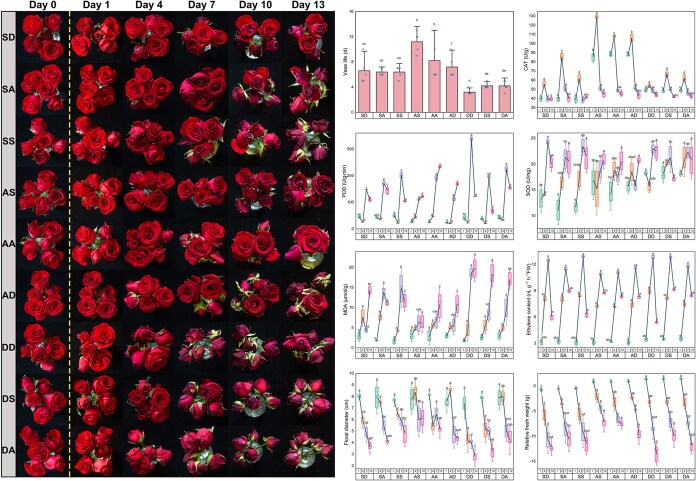
Top view of cut rose (*R. hybrida* L. cv. Carola) of different treatment groups on Day 0, 1, 4, 7, 10, and 13. The dotted line in the top view indicates that pulse treatment was performed here. Eight physiological parameters (vase life, CAT, POD, SOD, MDA, ethylene content, flower diameter, and RFW) of fresh cut rose in different treatment groups were determined on Day 1, 4, 7, and 10. One-way analysis of variance (ANOVA) was used to analyze the significance of differences in each parameter among the nine treatment groups on the same day, followed by Tukey’s honestly significant difference. Five replicates were used to test vase life, RFW, and floral diameter (*n* = 5). Three replicates were used to test the remaining indicators (*n* = 3). Box plots labeled with different letters indicate significant differences between treatment groups on the same day (*P* < 0.05). The box plots depict the mean (bold horizontal line), the upper and lower quartiles (boxes), and SD (whiskers). The mean of the box plots for different days for the same treatment group in each parameter were connected using the blue line.

Roses, one of the most popular ornamental cut flowers, were chosen for the validation experiment. Nine treatment groups were set up and exhibited in Table 1. Figure 6 shows the top view of cut roses (*Rosa hybrida* L. cv. Carola) and the results of eight physiological parameter measurements. The best preservation quality (from flower appearance performance) was observed in the group of 30 mg/l silver nanoparticles (AgNPs) pulse treatment and 2% sucrose solution as vase solution followed (AS). The vase life of fresh cut roses could be extended by sucrose pulse treatment groups (SD, SA, and SS) or AgNPs pulse treatment groups (AS, AA, and AD), among which the effect of 30 mg/l AgNPs pulse treatment was more pronounced (*P* < 0.05) than that of 5% sucrose pulse treatment.

**Table 1:** Nine groups set up by different pulse treatment and different vase solution treatment.

Group	Treatment	
	Pulse treatment	Vase solution treatment
SD	Sucrose (5%)	Distilled water
SA	Sucrose (5%)	AgNPs (2.5 mg/l)
SS	Sucrose (5%)	Sucrose (2%)
AS	AgNPs (30 mg/l)	Sucrose (2%)
AA	AgNPs (30 mg/l)	AgNPs (2.5 mg/l)
AD	AgNPs (30 mg/l)	Distilled water
DD	Distilled water	Distilled water
DS	Distilled water	Sucrose (2%)
DA	Distilled water	AgNPs (2.5 mg/l)

The name of group consists of two letters, with the first letter representing the preservative used in pulse treatment and the second letter representing the preservative used in vase solution treatment. Among them, ‘S’ stands for sucrose, ‘D’ stands for distilled water, and ‘A’ stands for AgNPs.

As can be seen from Fig. 6, the activities of antioxidant enzyme (CAT, POD, and SOD) in cut rose flowers exhibited a similar trend over time, which generally increased first and then decreased. CAT activities were significantly higher (*P* < 0.05) in the AgNPs pulse treatment groups (AS, AA, and AD) than those in the other groups on Days 1 and 4 and still had a higher level on Day 10. POD and SOD activities in the AgNPs pulse treatment groups (AS, AA, and AD) showed a general continuous upward trend, which was different from those in the other groups. There was no significant difference in the MDA contents of cut rose among the groups on Day 1 after treatment, but on Days 4, 7, and 10 the MDA contents of sucrose pulse treatment groups and AgNPs pulse treatment groups (SD, SA, SS, AS, AA, and AD) were generally lower than those of distilled water pulse treatment groups (DD, DS, and DA). At Day 10, MDA content was significantly lower (*P* < 0.05) in AS than those in the other groups. Comparing the trend of MDA content, it was found that pulse treatment reduced MDA accumulation, especially 30 mg/l AgNPs pulse treatment.

The ethylene content in each group also showed the trend of rising first and then decreasing. The ethylene contents of SA, AS, AA, AD, and DA were similar on the fourth and seventh days, but significantly lower than those of the other groups (*P* < 0.05). AS had the most effective mitigating effect on the reduction of flower diameter, and then SS and AA. When comparing the relative FW of the same group on Day 1 and Day 0, there were large differences in the changes of relative FW among different groups. Sucrose pulse treatment groups (SD, SA, and SS) showed a decrease in relative FW after pulse treatment with 5% sucrose solution compared to that before pulse treatment (Day 0), while the relative FWs on Day 1 were higher than those on Day 0 in AgNPs pulse treatment groups (AS, AA, and AD) and distilled water pulse treatment groups (DD, DS, and DA) (Fig. 6). Therefore, pulse treatment with 30 mg/l AgNPs could effectively delay the decline of FW of fresh cut roses (*R. hybrida* L. cv. Carola) during the preservation period.

## Discussion

Vase life, the most direct indicator of cut flower preservation efficacy, reflects preservative effectiveness through measurable physiological responses. Various factors influence vase life during postharvest preservation of fresh cut flowers, such as nutrient deficiencies, water/hormone imbalances, oxidative stress, and microbial invasion [12, 23, 24]. How to control these factors to prolong the vase life of cut flowers has become a guiding principle in the current development of preservatives. Carbohydrate-based preservatives primarily counteract postharvest nutrient depletion, while organic acids and inorganic compounds mitigate oxidative stress during senescence [25]. Exogenous phytohormones regulate endogenous hormonal balance to prolong vase life. Additionally, various nanomaterials have been reported to improve water balance and provide antimicrobial protection [12, 24].

### Pulse treatment has demonstrated superior efficacy in cut flower preservation compared to vase solution treatment

Meta-analysis results in this study, aligning with prior reports [26], demonstrated pulse treatment’s superior vase life extension compared to vase solutions (Fig. 1). This preservation efficacy was closely related to the activation of antioxidant enzymes induced by pulse treatment, which rapidly removed ROS and reduced oxidative damage (Fig. 1h). Through comparing the correlation of eight physiological parameters between pulse treatment and no pulse treatment, it was notable that the correlations between the changes of vase life and the changes of CAT/POD activities were negative in fresh cut flowers after pulse treatment, but positive in vase solution-treated flowers (Fig. 3). This phenomenon may be also closely related to the superior preservation efficacy of pulse treatment. With the increase of preservation time, ROS contents increase gradually in cut flowers, resulting in an activation in antioxidant enzymes rapidly. In the later preservation period, the antioxidant enzymes in cut flowers are consumed in large quantities and cannot be replenished continuously due to that cut flowers are separated from their parents, so the activities of antioxidant enzymes begin to decline. Thus, in the whole preservation stage of cut flowers, the antioxidant enzyme activities of cut flowers tend to show a trend of increasing firstly and then decreasing, which are slowed down by pulse treatment. It was found that, in the study of Wu *et al*., antioxidant enzyme activities of cut roses (*R. hybrida* L. cv. Carola) tended to increase and then decrease throughout the preservation process [12]. In addition, it was demonstrated that there was a slowing down of this process in the antioxidant enzymes of cut roses (*R. hybrida* L. cv. First Red) treated with pulses using AgNPs [27]. Our verification experiment results in Fig. 6 also validated the above phenomenon. Therefore, the linear relationship between antioxidant enzyme activity changes and vase life changes was highly dependent on whether antioxidant enzyme activity was in an ascending or descending phase, so it might show opposite trends between pulse treatment and vase solution treatment. Furthermore, pulse treatment more effectively suppressed ethylene accumulation in this study (Fig. 1g), contributing to their enhanced preservation efficacy. While floral diameter remained crucial for ornamental value assessment [28], pulse treatment showed limited effectiveness in this aspect due to their transient action, unlike vase solution treatment that maintained continuous exposure throughout the whole preservation period [29].

### The excellent preservation effect of nanomaterials was mainly achieved by the synergistic effect of water balance and antibacterial properties

Nanomaterials, widely investigated as novel preservatives [12, 13, 30], outperformed other agents in vase life extension (Fig. 1a) through distinct mechanisms—primarily maintaining water balance and inhibiting microbial growth rather than enhancing antioxidant enzyme activities [12]. Wu *et al*. proved that through the observation using stereomicroscope and scanning electron microscope, the nanomaterial graphene oxide adhered to the xylem at the stem end of cut flowers, reducing bacterial secretions that block the stem’s ducts and thus maintaining water balance of cut flowers [12]. Thus nanomaterials effectively mitigated water imbalance, and might significantly contribute to floral diameter maintenance through hydration regulation, although no studies had yet systematically investigated their impact on floral dimensions. The preservative of glycolic acid promoted the growth of microorganisms in the vase solution, which in turn reduced the water uptake of cut flowers [31], even resulted in water imbalance, while the use of nanomaterials could fill this problem effectively. Previous studies had confirmed AgNPs adhesion to microbial cells, penetration into the cell, production of ROS and free radicals, and modulation of microbial signaling pathways were considered to be the predominant antimicrobial modes of action [32]. The antimicrobial properties of nanomaterials were used as cut flower preservatives, e.g. graphene oxide extended vase life of cut roses (*R. hybrida* L. cv. Carola) by reducing bacterial levels in cutting rose preservation solution [11]. Figure 6 clearly also showed that the use of AgNPs as a preservative has an excellent preservation effect. It was worth noting that AgNPs also had a better effect on reducing the production of ethylene in cut flowers in both the pulse treatment and the vase solution treatment. Carnations are typical ethylene-sensitive cut flowers. AgNPs pulse treatment significantly reduced the ethylene content in their flowers. It is speculated that AgNPs inhibited the expression of positive regulatory factor genes in the ethylene signal transduction pathway of ethylene-sensitive cut flowers and suppressed their ethylene biosynthesis genes (e.g. *ACS1* and *ACO1*) [30]. In addition, current research has investigated the differences in mRNA levels of the ethylene-sensitive cut flower *R. hybrida* L. cv. Matador and the non-ethylene-sensitive cut flower *R. hybrida* L. cv. Dolcetto in response to exogenous ethylene following AgNPs treatment [13]. The downregulation of ethylene-induced receptor genes (*RhETR2*, *RhETR3*, and *RhETR5*) was effectively suppressed in *R. hybrida* L. cv. Matador on Days 4 and 6, whereas the suppression was not observed in *R. hybrida* L. cv. Dolcetto [13]. However, there has been little research on the molecular mechanisms of pulse treatment of ethylene-sensitive and non-ethylene-sensitive cut flowers using nanomaterials, which warrants further investigation. The limited effect of nanomaterials on relative FW retention (Fig. 1b) might be due to their inability to supplement the depletion of respiratory substrates in fresh cut flowers [33]. Therefore, the application of nanomaterials as preservatives should incorporate nutrient supplementation to compensate for respiratory substrate depletion during cut flower metabolism.

### Plant species-specific preservation should be considered

For Lamiales species, preservative treatment showed no significant impact on vase life but induced marked MDA accumulation (Fig. 1a and h). The observed results might stem from either the unsuitability of current preservation methods for Lamiales or the limited number of studies conducted on this taxon, highlighting the need for more extensive investigation. Although Rosales had been extensively studied [34, 35], few studies had examined its antioxidant enzyme responses, likely due to predominant research focus on antibacterial effect of preservatives in this species [26, 36]. So the same preservative exhibited varying efficacy in maintaining freshness across different cut flower species. Carlson *et al*. also demonstrated the same results as above [37]. One determinant of flower senescence was ethylene sensitivity: ethylene-dependent species showed senescence regulated by ethylene signaling, while ethylene-independent species exhibited minimal ethylene response [38]. In addition, the ability of sensing ethylene changed during flower development, which might lead to differences in the sensitivity to ethylene in different flower species [39]. In *et al*. found that rapid accumulation of ACO (1-aminocyclopropane-1-carboxylic acid oxidase) in cut rose flowers of ethylene-sensitive varieties under ethylene exposure led to a significant shortening of vase life and was strongly correlated with the level of ethylene-induced transcripts of *RhACO1* (one of the ethylene biosynthesis genes), but the vase life of ethylene-insensitive varieties was unaffected by ethylene treatment [40]. Figure 1g showed that the ethylene content variation of Liliflorae did not respond significantly to the studied preservatives. In the study of Hassan *et al*., gladiolus (one species of Liliflorae) cut flowers were considered to be ethylene-insensitive cut flowers, despite minor ethylene fluctuations during preservation, the treated flowers maintained ethylene levels comparable to controls at the end of the preservation period [41], contrasting sharply with ethylene-sensitive species like carnations (belonging to Centrospermae) [30]. By determining whether a cut flower is ethylene-sensitive or non-ethylene-sensitive, it is possible to select a more effective preservation substance for vase solution treatment. 1-methylcyclopropene (1-MCP) has long been used as an ethylene-sensing inhibitor and has been shown to exhibit excellent effects on the preservation of ethylene-sensitive cut flowers [42], but the current reports have found that different rose genotypes influence the action duration of 1-MCP [43]. Therefore, even different species belonging to the cut rose category need to adapt their preservation strategies for different varieties. The article also stated that the use of 1-MCP as a method of preservation should be avoided for non-ethylene-sensitive cut flowers [43]. Glycolic acid, an organic acid, with demonstrated preservative efficacy in cut flower preservation [44], but promoted bacterial proliferation in vase solutions. When glycolic acid was applied to Hydrangea preservation, it resulted in accelerated vase life reduction [31]. This contradictory phenomenon might arise from Hydrangea’s heightened susceptibility to phytopathogenic fungi. It also possible that Hydrangea was infected at an early stage of development and developed symptoms quickly after harvest [45]. Cut flowers that were susceptible to attack by pathogenic microorganisms should be preserved with a strong antimicrobial preservation. Thus, different preservation mechanisms have emerged for different species of flower, which highlights the need for plant species-specific preservation strategies.

### An optimized preservative strategy was proposed by integrating the advantages of pulse treatment and nanomaterials and the differences in plant species.

Recent studies have increasingly explored multipreservative combinations for cut flower preservation [46, 47], yet a theoretical protocol for rational preservative application remains absent. In this study, experimental data from 45 published literatures was used for model construction, and the best fitting models were used to predict the missing data in the measured parameters. The R^2^ values were found >0.6 in most prediction models (Fig. 4), and a distinction was observed between pulse treatments and vase solution treatments based on the results of t-SNE analysis (Fig. 5). Although Campanulales exhibited minimal responsiveness to pulse treatment, highlighting the need to classify plant species by pulse treatment sensitivity for tailored preservation protocols. As for preservative types, there was differentiation between pulse treatment and vase solution treatment, indicating that pulse treatment could amplify the preservation efficacy of preservatives, which might be due to distinct preservative mechanisms depending on treatment methods.

Pulse treatment could extend the vase life of cut flowers more than vase solution treatment. Due to their structural properties, nanomaterials had the superior properties of maintaining water balance and inhibiting bacteria growth of cut flowers. Therefore, the optimal preservation strategy was proposed based on the above findings: nanomaterials used in pulse treatments could better exploit their special preservation efficacy, subsequently, based on plant species, appropriate preservatives were selected for vase solution to provide continuous nutritional support and signal stimulation. The above preservation strategy could use pulse treatment to maximize the advantages of nanomaterials. Our small-scale verification experiment results (Fig. 6) showed that the preservation effect of AS (30 mg/l AgNPs pulse treatment followed by 2% sucrose as vase solution treatment) on cut rose (*R. hybrida* L. cv. Carola) was significantly better than that of other treatment groups, which was excellent evidence of the effectiveness of this above preservation strategy.

## Conclusion

Through systematic analysis of 45 published studies on cut flower preservation, this investigation yielded three pivotal conclusions: (i) Pulse treatment demonstrated substantial advantages over vase solution treatment in vase life extension, which was due to its completely different preservation mechanism from vase solution treatment. However, its transient nature limited the capacity to maintain floral appearance, therefore pulse treatment followed by vase solution treatment was the best option for freshness of cut flowers. (ii) Nanomaterials exhibited unique preservation benefiting through water balance and antimicrobial action, complementing other preservative types that primarily enhanced antioxidant activity. Their inability to provide nutritional support necessitated synergistic applications with conventional preservatives. (iii) Plant species-specific preservation strategies must consider both preservative type and application method based on taxon characteristics (e.g. ethylene sensitive or insensitive).

Based on these findings, an optimized preservation protocol was proposed: applying nanomaterials through pulse treatment, followed by plant species-specific vase solution to provide subsequent nutritional support. Such optimized preservation strategy combined the advantages of nanomaterials and pulse treatment, while the subsequent plant species-specific vase solution treatment compensated for the nutritional deficiencies of nanomaterials, which might enhance the vase life of cut flowers beyond existing methodologies. Small-scale validation experiments have demonstrated that pulse treatment helps extend the vase life of cut roses (*R. hybrida* L. cv. Carola), and that combining pulse treatment with vase solution is more effective than using either treatment alone. However, different flower varieties have varying requirements for the types, concentrations, and treatment times of preservatives. Additionally, the chemicals and treatment conditions used in the validation experiments were only set at moderate levels, and the most suitable combinations or conditions still require further in-depth research.

## Materials and method

### Literature collection and study protocol

In this study, published research papers were collected from the Web of science (WOS) database using the search terms TS = (‘cut flower’ OR ‘vase life’) AND TS = (‘preservation’). We searched for article titles, abstracts, and keywords, including 2588 research papers up to 17 August 2024, the research steps shown in Fig. S3a were performed. (i) Papers related to this study (*n* = 180) were retained after reading the articles. (ii) Articles with inaccessible full texts or insufficient data for extraction were excluded from the analysis, and the articles containing available experimental data were retained (*n* = 103). (iii) The studies that matched the research requirements of this paper were retained (*n* = 45), e.g. the articles that did not set up control group using clear water and used a variety of substances as preservatives within the article were filtered out. (iv) Finally, 45 research papers were screened and then subjected to meta-analysis, correlation analysis, and construction of machine learning models.

### Meta-analysis

#### Data extraction

The experiment results from the 45 research papers screened were used for meta-analysis (Table S1). Eight key parameters in fresh cut flower preservation, including vase life, relative FW, floral diameter, ethylene content, SOD activity, CAT activity, POD activity, and MDA content, were selected. The data in the article graphs were obtained using Origin software (version 9.8.0.200). The data from the 45 articles were categorized based on four influencing factors: Pulse treatment (‘YES’ represents that pulse treatment was performed, and ‘NO’ represents that no pulse treatment was performed), plant species (divided into Campanulales, Liliflorae, Centrospermae, Ranales, Lamiales, and Rosales), preservative types (Inorganic compound, Plant growth regulators and hormones, Nanomaterials, and Carbohydrate and Organic acids), and concentration (‘Low’ represents a preservative concentration of ≤0.00002%, ‘High’ represents a preservative concentration of >0.00002%.). For experiments with multiple measurement days, only data measured on the last day were included in this experiment. If the data provided in the investigated article only included the FW of cut flowers on each day and did not calculate the relative FW, the experiment was conducted using the FW measured on the first day minus the data measured on the last day. If the standard deviation (SD) was not provided in the article, it was calculated as 10% of the mean.

#### Meta-analysis

Meta-analysis was performed using the *meta* software package in R 4.0.3. The detailed description of the formulae is provided in Text S1. In this study, the fresh cut flowers cultured without preservative were used as control group and the fresh cut flowers cultured with preservative were used as treatment group. Meta-analysis was performed using log response ratio (lnRR) [17, 48], which was converted to effect size for ease of interpretation to plot forest plots with 95% confidence intervals (CI) [49, 50]. Begger’s test was used to assess whether there was publication bias for the eight physiological parameters (Table S2), and the results showed that there was no significant publication bias (*P >* 0.05).

### Relevance analysis

Pearson’s correlation analysis was performed using the *scipy* package in Python 3.11 for each physiological parameter (Text S2). In addition, ChiPlot (https://www.chiplot.online/) was used to analyze the linear relationships between physiological parameters, and linear relationships between physiological parameters were also analyzed based on the categories of different influencing factors, such as plant species, preservative types, and pulse treatment.

### Machine learning

The physiological parameters were scattered in the current 45 papers, which produce difficulty in the subsequent study. Machine learning was used to construct a series of models of lnRR for the eight physiological parameters, and the best performing models, with the highest value of *R*^2^, were selected to predict the missing data for each physiological parameter. In this experiment, machine learning models of lnRR for each physiological parameter were constructed using Python 3.11.

#### Inclusion of characteristic variables

In the training of the model, various feature variables were considered, including plant species, preservative types, whether pulse treatment, preservative concentration. Additionally, the lnRR values of vase life and relative FW, which have been widely studied, were also incorporated into the model construction of the physiological parameters (except vase life and relative FW themselves), contributing to the enhancement of model predictive capability.

#### Model selection

In this experiment, 11 models for each physiological parameter were selected for training and their results were compared (Table S3). The selected models included Random Forest (RF), eXtreme Gradient Boosting (XG), Support Vector Regression (SVR), Artificial Neural Network (ANN), K-Nearest Neighbors (KNN), Kernel Regression (KRR), Gradient Boosting (GBT), Linear Regression (LR), Ridge Regression (Ridge), Quadratic Polynomial Regression (PR_2), and Cubic Polynomial Regression (PR_3).

#### Training models

Numerical features of all data set were *Z*-score normalized prior to constructing the model. In order to improve the predictive ability of the models, the feature variables were exhaustively enumerated to construct the model, aiming to filter the combination of feature variables with *R*^2^ and mean square error (MSE) under the same model. Each model was evaluated by Leave-One-Out Cross-Validation (LOOCV), holding out one sample per fold. Within each leave-one-out fold, every model was always trained on the entire available training set (all samples except the one held out). The ANN model ran up to 500 epochs, and reserved 10% of each training fold for early stopping. XG and RF models each built 500 trees in a single fit, and the GBT model built 200 trees in a single fit. LR, Ridge, and PR models were solved in closed form in one shot, without batches or iterations. The model and feature combination with the best R^2^ and MSE were selected for the subsequent study.

#### Model prediction and t-SNE analysis

The predicted filled dataset was obtained by supplementing the missing lnRR of the metrics separately by the constructed model. In order to ensure the prediction accuracy, only the articles containing vase life data were filled in, so only one article lacking vase life data was excluded. The predicted plant species, pulse treatment, preservative types, and their correspondence with each other were visualized and given in Fig. S3b, and then the completed dataset was analyzed by t-SNE analysis.

### Validation experiment

#### Experimental group settings

Nine treatment groups were set up based on different pulse treatments and vase solution treatments (Table 1). The concentrations of AgNPs and sucrose employed in the pulse and vase solution treatments were based either on previously reported levels demonstrating significant preservative efficacy or on optimal concentrations established in preliminary experimental studies [27, 50, 51]. The AgNPs used in the experiments were purchased from Pengrui Nano Technology Ltd., China. Fresh cut roses (*R. hybrida* L. cv. Carola) were purchased from a local flower market in Tianjin, China, and immediately delivered to the laboratory. This variety of rose is a typical ethylene-sensitive variety [52]. Cut roses of similar growth and openness were selected, the stem ends were cut diagonally at 45 degrees, and all leaves removed. The stems of treated cut roses were rinsed with distilled water and then pulse-treated for 24 h. The preservation treatment was conducted at a temperature of 23°C–26°C and a relative humidity of 18%–38%. On Day 6 of the experiment, vase solution with corresponding concentration for each treatment group was updated.

#### Vase life and relative fresh weight of cut roses

Vase life is defined as the number of days that cut flowers are preserved from pulse treatment (Day 0) until 50% of the petals wilted (petals shrivel or discolor) or bent neck is observed. The FWs of cut roses were measured on Days 0, 1, 4, 7, and 10 and the photographs of each treatment group were taken with a digital camera. Five cut flowers per group were used to count vase life as well as relative FW measurements.

The FW of the cut roses was measured during the observation period to calculate the relative fresh weight (RFW) using the formula:


$$ \mathrm{RFW}\ \left(\mathrm{g}\right)={\mathrm{FW}}_d\hbox{--} {\mathrm{FW}}_0. $$

Where FW_d_ was the FW of the cut roses (g) on *d* = Day 1, 4, 7, 10, and FW_0_ was the weight of the cut roses (g) on Day 0.

#### Antioxidant enzyme activity and MDA content measurements

Variation in antioxidant enzyme activities in cut flowers was assessed by the changes in POD, SOD, and CAT activities, and membrane lipid peroxidation in cut flowers was assessed by the content of MDA. Petals of cut roses were randomly selected for antioxidant enzyme studies on Days 1, 4, 7, and 10 after the start of the experiment. POD activity was determined by guaiacol method [53], the increase in absorbance at 470 nm was measured, and POD activity was expressed in U/g·min. SOD activity, defined as the amount of enzyme causing 50% inhibition of nitroblue-tetrazolium (NBT) reduction at 560 nm, was determined according to the method of Wu *et al*. and expressed in U/mg. CAT activity was determined by monitoring the decrease in absorbance at 240 nm due to H_2_O_2_ degradation in U/g [54]. MDA content in cut flower petals was determined by trichloroacetic acid (TCA) and thiobarbituric acid (TBA) methods [55]. Three independent replicates per group were set up.

#### Ethylene content measurement

Fresh cut rose flower samples were collected on Days 1, 4, 7, and 10, with three replicates of five cut flowers per treatment group, placed in an airtight container, and incubated at 25°C for 120 min. A sample of headspace gas was then taken with a gas-tight syringe and injected into a gas chromatograph (7890B GC, Agilent, USA) [56] for ethylene concentration measurement. Ethylene content was expressed as (nl g^−1^ h^−1^ FW).

#### Flower diameter measurement

Flower diameter of fresh cut flowers was measured with a Vernier caliper on Days 1, 4, 7, and 10. Five cut flowers were measured in each treatment group at each time point.

#### Statistical analysis

The data obtained were subjected to computation of mean, SD, one-way ANOVA and Tukey’s honestly significant difference using the Statistical Package for Social Sciences (SPSS) version 26.0 and box plotting using Origin software (version 9.8.0.200).

## Supplementary Material

Web_Material_uhaf227

## Data Availability

All the data underlying this article are available within the article and its online supplementary material.
