# Supplementary material for: Postharvest preservation efficacy and optimization strategies of fresh cut flowers: a meta-analysis and machine learning approach
Source: Hortic Res. 2025 Sep 3;12(12):uhaf227. doi: 10.1093/hr/uhaf227 (PMC12680378; doi:10.1093/hr/uhaf227)
Supplement: Web_Material_uhaf227 [file web_material_uhaf227.zip › Supplmentary information20250623.docx]

**Text S1.** Statistical analysis of meta-analysis.

Meta-analysis was performed using the “*meta*” package in R 4.0.3.

The lnRR was calculated as follows, with the group to which only clean water was applied set as the control group (control) and the group to which one type preservative applied defined as the treatment group (treat). *X_c_* is the mean of each physiological parameter in the control group and *X_t_* is the mean of each physiological parameter in the treatment group.

lnRR = ln$(\frac{X_{t}}{X_{c}})$ = ln (*X_t_*) - ln (*X_c_*) (1)

$N_{c}$ and $N_{t}$ denote the sample size of the control and treatment groups, respectively, $S_{c}$ and $S_{t}$ denote the standard deviations of the control and treatment groups, respectively, $v_{i}$ denotes the within-study variance, calculated as follows:

$v_{i}$ = $\frac{\left( S_{t} \right)^{2}}{{N_{t}(X_{t})}^{2}}$ + $\frac{\left( S_{c} \right)^{2}}{{N_{c}(X_{c})}^{2}}$ (2)

$\sigma^{2}$ denotes the between-studies variance and Q denotes the inter-experimental variance component estimates:

*w_i_ =* $\frac{1}{v_{i}}$ (3)

Q = $\sum_{i=1}^{k} w_{i}\left( \mathrm{lnRR}_{i} \right)$ – $\frac{\left( \sum_{i=1}^{k} w_{i}\mathrm{lnRR}_{i} \right)^{2}}{\sum_{i=1}^{k} w_{i}}$ (4)

$\sigma^{2}$ = $\frac{Q - (k-1)}{\sum_{i=1}^{k} w_{i} - \frac{\sum_{i=1}^{k} {w_{i}}^{2}}{\sum_{i=1}^{k} w_{i}}}$ (5)

$w_{i}^{*}$ denotes the weighting factor in the experimental group, which is calculated as follows:

$w_{i}^{*}$ = $\frac{1}{v_{i} + \sigma^{2}}$ (6)

$\mathrm{lnRR}^{*}$ denotes the weighted mean ratio rate of each physiological parameter:

$\mathrm{lnRR}^{*}$ = $\frac{\sum_{i=1}^{k} w_{i}^{*}\mathrm{lnRR}_{i}}{\sum_{i=1}^{k} w_{i}^{*}}$ (7)

The standard error of $\mathrm{lnRR}^{*}$ is calculated as follows:

$v_{i}^{*}$ = $\sqrt{\frac{1}{\sum_{i=1}^{k} w_{i}^{*}}}$ (8)

The 95 % confidence interval (CI) for $\mathrm{lnRR}^{*}$:

95%CI = $\mathrm{lnRR}^{*}$ ± 1.96*s*($\mathrm{lnRR}^{*}$) (9)

Convert the size effect to a percentage form:

C = ($e^{\mathrm{lnRR}^{*}}$ - 1) $\times$ 100%. (10)

**Text S2.** Pearson's correlation analysis of lnRR.

Pearson's correlation analysis was performed using the “*scipy*” package in Python 3.11.

Respectively, *X_i_* and *Y_i_* are the sample values of the two variables; $\bar{X}$ and $\bar{Y}$ are the means of the samples of the two variables. *n* is the sample size.

r = $\frac{\sum_{i=1}^{n} (X_{i}-\bar{X})(Y_{i}-\bar{Y})}{\sqrt{\sum_{i=1}^{n} {(X_{i}- \bar{X})}^{2}} \cdot\sqrt{\sum_{i=1}^{n} {(Y_{i}- \bar{Y})}^{2}}}$ (1)

The value of r ranges from [-1, 1], a positive value indicates a positive correlation between the two variables, a negative value indicates a negative correlation between the two, and a value of 0 indicates that there is no linear correlation between the two.

*P* < 0.05 indicates significant correlation, and *P* < 0.01 indicates highly significant correlation.

**Table S1.** The 45 articles selected for meta-analysis and machine learning.

| **Title** | **Journal** | **DOI** | **Public year** |
| --- | --- | --- | --- |
| Hydrogen Nanobubble Water Delays Petal Senescence and Prolongs the Vase Life of Cut Carnation (*Dianthus caryophyllus* L.) Flowers | PLANTS-BASEL | 10.3390/plants10081662 | 2021 |
| Sodium selenite improves the vase life of *Eustoma grandiflorum* cut flowers | HORTICULTURAL SCIENCE | 10.17221/61/2021-HORTSCI | 2022 |
| Hydrogen gas increases the vase life of cut rose 'Movie star' by regulating bacterial community in the stem ends | POSTHARVEST BIOLOGY AND TECHNOLOGY | 10.1016/j.postharvbio.2021.111685 | 2021 |
| Vase life extension of cut hydrangea (*Hydrangea macrophylla*) flowers | JOURNAL OF HORTICULTURAL SCIENCE & BIOTECHNOLOGY | 10.1080/14620316.2019.1676660 | 2020 |
| Effect of hydrogen-rich water on vase life and quality in cut lily and rose flowers | HORTICULTURE ENVIRONMENT AND BIOTECHNOLOGY | 10.1007/s13580-017-0043-2 | 2017 |
| Hydrogen Sulfide Improves the Vase Life and Quality of Cut Roses and Chrysanthemums | JOURNAL OF PLANT GROWTH REGULATION | 10.1007/s00344-021-10312-7 | 2021 |
| Cerium improves the vase life of *Dianthus caryophyllus* cut flower by regulating the ascorbate and glutathione metabolism | SCIENTIA HORTICULTURAE | 10.1016/j.scienta.2018.06.046 | 2018 |
| Cerium improves the vase life of *Lilium longiflorum* cut flowers through ascorbate-glutathione cycle and osmoregulation in the petals | SCIENTIA HORTICULTURAE | 10.1016/j.scienta.2017.09.040 | 2018 |
| Lanthanum delays the senescence of *Lilium longiflorum* cut flowers by improving antioxidant defense system and water retaining capacity | SCIENTIA HORTICULTURAE | 10.1016/j.scienta.2015.10.012 | 2015 |
| ClO_2_ Prolongs the Vase Life of *Paeonia lactiflora* 'Hushui Dangxia' Cut Flowers by Inhibiting Bacterial Growth at the Stem Base | HORTICULTURAE | 10.3390/horticulturae10070732 | 2024 |
| Characterization of the role of sodium nitroprusside (SNP) involved in long vase life of different carnation cultivars | BMC PLANT BIOLOGY | 10.1186/s12870-017-1097-0 | 2017 |
| Effect of gibberellin A_3_ on leaf yellowing and vase life of cut *Narcissus tazetta* var. *chinensis* flowers | JOURNAL OF THE JAPANESE SOCIETY FOR HORTICULTURAL SCIENCE | none | 2000 |
| 1-MCP improves the postharvest quality of cut spray carnation (*Dianthus caryophyllus* L.) 'Optima' flowers | HORTICULTURE ENVIRONMENT AND BIOTECHNOLOGY | 10.1007/s13580-013-0044-8 | 2013 |
| Action of abscisic and gibberellic acids on senescence of cut gladiolus flowers | BRAGANTIA | 10.1590/1678-4499.361 | 2016 |
| Polyglutamic acid as a vase life improver for cut lilies | HORTICULTURAL SCIENCE | 10.17221/148/2022-HORTSCI | 2023 |
| The involvement of NO in ABA-delayed the senescence of cut roses by maintaining water content and antioxidant enzymes activity | SCIENTIA HORTICULTURAE | 10.1016/j.scienta.2018.12.006 | 2019 |
| Effect of gibberellic acid on the vase life and oxidative activities in senescing cut gladiolus flowers | PLANT GROWTH REGULATION | 10.1007/s10725-013-9839-y | 2014 |
| Protective effects of 1-methylcyclopropene and salicylic acid on senescence regulation of gladiolus cut spikes | SCIENTIA HORTICULTURAE | 10.1016/j.scienta.2014.09.025 | 2014 |
| Neoagaro-oligosaccharides Improve the Postharvest Flower Quality and Vase Life of Cut Rose 'Gaoyuanhong' | HORTSCIENCE | 10.21273/HORTSCI16988-22 | 2023 |
| Effect of green tea extract powder on the vase-life of fresh-cut rose (*Rosa hybrida* L.) 'Carola' stems | JOURNAL OF HORTICULTURAL SCIENCE & BIOTECHNOLOGY | 10.1080/14620316.2016.1155316 | 2016 |
| Effects of Different Preservatives on Cut Flower of *Luculia pinceana*: A Novel Fragrant Ornamental Species | HORTSCIENCE | 10.21273/HORTSCI15724-21 | 2021 |
| Effects of Trehalose and Sucrose on the Vase Life and Physiology of Cut Astilbe (*Astilbe* x *arendsii* Arends) Flowers | HORTICULTURE JOURNAL | 10.2503/hortj.UTD-031 | 2019 |
| Effect of salicylic and ascorbic acids on post-harvest vase life of Chrysanthemum cut flowers | HORTICULTURAL SCIENCE | 10.17221/151/2020-HORTSCI | 2022 |
| Citric acid, sucrose and Cu^2+^ as potential vase treatments for cut *Acacia holosericea* G. Don foliage stems | JOURNAL OF HORTICULTURAL SCIENCE & BIOTECHNOLOGY | 10.1080/14620316.2017.1344570 | 2018 |
| Antioxidative activities and qualitative changes in gladiolus cut flowers in response to salicylic acid application | SCIENTIA HORTICULTURAE | 10.1016/j.scienta.2016.07.034 | 2016 |
| Ascorbic Acid Increases Cut Flower Longevity of Sword Lily by Regulating Oxidative Stress and Reducing Microbial Load | JOURNAL OF PLANT GROWTH REGULATION | 10.1007/s00344-024-11396-7 | 2024 |
| Acetylsalicylic acid increases postharvest longevity of ornamental sunflower inflorescence | SEMINA-CIENCIAS AGRARIAS | 10.5433/1679-0359.2021v42n3Supl1p1411 | 2021 |
| Salicylic acid and sodium nitroprusside improve postharvest life of chrysanthemums | SCIENTIA HORTICULTURAE | 10.1016/j.scienta.2012.07.016 | 2012 |
| Delaying petal and leaf senescence in Yellow Star chrysanthemum using ascorbic acid | INDIAN JOURNAL OF HORTICULTURE | 10.5958/0974-0112.2018.00085.3 | 2018 |
| Chitooligosaccharide Prolongs Vase Life of Cut Roses by Decreasing Reactive Oxygen Species | KOREAN JOURNAL OF HORTICULTURAL SCIENCE & TECHNOLOGY | 10.7235/hort.2015.14188 | 2015 |
| The combination of graphene oxide and preservatives can further improve the preservation of cut flowers | FRONTIERS IN PLANT SCIENCE | 10.3389/fpls.2023.1121436 | 2023 |
| A novel efficient multi-walled carbon nanotubes/gibberellic acid composite for enhancement vase life and quality of *Rosa hybrida* cv. 'Moonstone' | BMC PLANT BIOLOGY | 10.1186/s12870-024-04925-9 | 2024 |
| Nano-silver controls transcriptional regulation of ethylene- and senescence-associated genes during senescence in cut carnations | SCIENTIA HORTICULTURAE | 10.1016/j.scienta.2021.110280 | 2021 |
| Synergistic Effect of Nano-Sliver with Sucrose on Extending Vase Life of the Carnation cv. Edun | FRONTIERS IN PLANT SCIENCE | 10.3389/fpls.2017.01601 | 2017 |
| Nanosilver and sucrose delay the senescence of cut snapdragon flowers | POSTHARVEST BIOLOGY AND TECHNOLOGY | 10.1016/j.postharvbio.2020.111165 | 2020 |
| Combined Nano Silver, α-Aminoisobutyric Acid, and 1-Methylcyclopropene Treatment Delays the Senescence of Cut Roses with Different Ethylene Sensitivities | HORTICULTURAE | 10.3390/horticulturae8060482 | 2022 |
| Effects of Pulse Treatments with Sucrose, Silver Thiosulfate Complex (STS) and Calcium Chloride on the Vase Life and Soluble Carbohydrate and Aurone Levels in Cut Snapdragon Flowers | HORTICULTURE JOURNAL | 10.2503/hortj.UTD-304 | 2022 |
| Effects of silver thiosulfate complex (STS), sucrose and their combination on the quality and vase life of cut *Eustoma* flowers | JOURNAL OF THE JAPANESE SOCIETY FOR HORTICULTURAL SCIENCE | 10.2503/jjshs.74.381 | 2005 |
| Nano-silver pretreatment delays wilting of cut gardenia foliage by inhibiting bacterial xylem blockage | SCIENTIA HORTICULTURAE | 10.1016/j.scienta.2018.11.050 | 2019 |
| Effects of three different nano-silver formulations on cut *Acacia holosericea* vase life | POSTHARVEST BIOLOGY AND TECHNOLOGY | 10.1016/j.postharvbio.2011.11.005 | 2012 |
| Plant growth regulators preserved the longevity of cut stems of *Chrysanthemum morifolium* by orchestrating physio-biochemical and anatomical responses | PLANT PHYSIOLOGY AND BIOCHEMISTRY | 10.1016/j.plaphy.2023.02.044 | 2023 |
| Salicylic acid modulates cutting-induced physiological and biochemical responses to delay senescence in two gerbera cultivars | PLANT GROWTH REGULATION | 10.1007/s10725-018-0466-5 | 2019 |
| Physiological and biochemical modifications by postharvest treatment with sodium nitroprusside extend vase life of cut flowers of two gerbera cultivars | POSTHARVEST BIOLOGY AND TECHNOLOGY | 10.1016/j.postharvbio.2017.11.009 | 2018 |
| Neoagaro-oligosaccharides Improve the Postharvest Flower Quality and Vase Life of Cut Rose 'Gaoyuanhong' | HORTSCIENCE | 10.21273/HORTSCI16988-22 | 2023 |
| Effect of green tea extract powder on the vase-life of fresh-cut rose (*Rosa hybrida* L.) 'Carola' stems | JOURNAL OF HORTICULTURAL SCIENCE & BIOTECHNOLOGY | 10.1080/14620316.2016.1155316 | 2016 |

**Table S2.** Begg's test for publication bias detection for each physiological parameter.

|  | Vase life | Ethylene content | MDA | CAT | Relative fresh weight | Floral diameter | POD | SOD |
| --- | --- | --- | --- | --- | --- | --- | --- | --- |
| *P* | 0.43 | 0.21 | 0.19 | 0.50 | 0.08 | 0.44 | 0.34 | 0.81 |

**Table S3.** Models used for prediction and their best coefficient of determination (R^2^) and mean square error (MSE). The rows filled in yellow were the best performing models for the 8 physiological parameters.

|  | R² | MSE | RMSE | Moudel type |
| --- | --- | --- | --- | --- |
| Vase life | 0.585612 | 0.040374 | 0.200932 | RF |
| Vase life | 0.538157 | 0.044997 | 0.212125 | XG |
| Vase life | 0.206593 | 0.077301 | 0.278031 | LR |
| Vase life | 0.475501 | 0.051102 | 0.226057 | PR_2 |
| Vase life | 0.159192 | 0.08192 | 0.286216 | PR_3 |
| Vase life | 0.512806 | 0.047467 | 0.21787 | Ridge |
| Vase life | 0.633029 | 0.035754 | 0.189087 | ANN |
| Vase life | 0.568076 | 0.042082 | 0.20514 | SVR |
| Vase life | 0.555783 | 0.04328 | 0.208038 | KNN |
| Vase life | 0.563989 | 0.04248 | 0.206108 | GBT |
| Vase life | 0.486161 | 0.050063 | 0.223748 | KRR |
| MDA | 0.54531 | 0.039 | 0.197484 | RF |
| MDA | 0.383165 | 0.052907 | 0.230016 | XG |
| MDA | 0.495532 | 0.043269 | 0.208013 | LR |
| MDA | 0.334222 | 0.057105 | 0.238967 | PR_2 |
| MDA | 0.306666 | 0.059469 | 0.243862 | PR_3 |
| MDA | 0.518367 | 0.041311 | 0.20325 | Ridge |
| MDA | 0.603312 | 0.034025 | 0.184458 | ANN |
| MDA | 0.607777 | 0.033642 | 0.183417 | SVR |
| MDA | 0.561599 | 0.037603 | 0.193914 | KNN |
| MDA | 0.522523 | 0.040954 | 0.202372 | GBT |
| MDA | 0.562001 | 0.037568 | 0.193825 | KRR |
| POD | 0.520141 | 0.06417 | 0.253318 | RF |
| POD | 0.498816 | 0.067022 | 0.258885 | XG |
| POD | 0.38673 | 0.082011 | 0.286375 | LR |
| POD | 0.628282 | 0.049709 | 0.222954 | PR_2 |
| POD | 0.4114 | 0.078712 | 0.280556 | PR_3 |
| POD | 0.623608 | 0.050334 | 0.224352 | Ridge |
| POD | 0.434345 | 0.075643 | 0.275033 | ANN |
| POD | 0.622125 | 0.050532 | 0.224793 | SVR |
| POD | 0.579497 | 0.056233 | 0.237134 | KNN |
| POD | 0.469479 | 0.070945 | 0.266355 | GBT |
| POD | 0.567175 | 0.05788 | 0.240583 | KRR |
| CAT | 0.541464 | 0.069481 | 0.263592 | RF |
| CAT | 0.556603 | 0.067186 | 0.259204 | XG |
| CAT | 0.438279 | 0.085116 | 0.291746 | LR |
| CAT | 0.523829 | 0.072153 | 0.268612 | PR_2 |
| CAT | 0.482652 | 0.078392 | 0.279986 | PR_3 |
| CAT | 0.53421 | 0.07058 | 0.265668 | Ridge |
| CAT | 0.509108 | 0.074383 | 0.272733 | ANN |
| CAT | 0.623075 | 0.057114 | 0.238986 | SVR |
| CAT | 0.597828 | 0.06094 | 0.24686 | KNN |
| CAT | 0.496851 | 0.076241 | 0.276117 | GBT |
| CAT | 0.590301 | 0.06208 | 0.249159 | KRR |
| SOD | 0.708173 | 0.042718 | 0.206682 | RF |
| SOD | 0.687245 | 0.045781 | 0.213965 | XG |
| SOD | 0.653491 | 0.050722 | 0.225215 | LR |
| SOD | 0.603941 | 0.057975 | 0.24078 | PR_2 |
| SOD | 0.629969 | 0.054165 | 0.232734 | PR_3 |
| SOD | 0.670271 | 0.048266 | 0.219694 | Ridge |
| SOD | 0.629833 | 0.054185 | 0.232777 | ANN |
| SOD | 0.705956 | 0.043042 | 0.207466 | SVR |
| SOD | 0.738551 | 0.038271 | 0.195629 | KNN |
| SOD | 0.678413 | 0.047074 | 0.216965 | GBT |
| SOD | 0.684552 | 0.046175 | 0.214884 | KRR |
| Ethylene content | 0.757047 | 0.780929 | 0.883702 | RF |
| Ethylene content | 0.767857 | 0.746185 | 0.86382 | XG |
| Ethylene content | 0.620654 | 1.219342 | 1.104238 | LR |
| Ethylene content | 0.858486 | 0.454874 | 0.674443 | PR_2 |
| Ethylene content | 0.630211 | 1.188623 | 1.09024 | PR_3 |
| Ethylene content | 0.627795 | 1.196389 | 1.093796 | Ridge |
| Ethylene content | 0.702387 | 0.956626 | 0.978073 | ANN |
| Ethylene content | 0.587666 | 1.325376 | 1.15125 | SVR |
| Ethylene content | 0.71178 | 0.926432 | 0.962513 | KNN |
| Ethylene content | 0.716301 | 0.911901 | 0.954935 | GBT |
| Ethylene content | 0.625925 | 1.202398 | 1.096539 | KRR |
| Floral diameter | 0.241507 | 0.036015 | 0.189777 | RF |
| Floral diameter | 0.300755 | 0.033202 | 0.182214 | XG |
| Floral diameter | 0.186026 | 0.03865 | 0.196595 | LR |
| Floral diameter | 0.605367 | 0.018738 | 0.136887 | PR_2 |
| Floral diameter | 0.107353 | 0.042385 | 0.205876 | PR_3 |
| Floral diameter | 0.56957 | 0.020438 | 0.142961 | Ridge |
| Floral diameter | 0.472265 | 0.025058 | 0.158298 | ANN |
| Floral diameter | 0.500035 | 0.02374 | 0.154077 | SVR |
| Floral diameter | 0.219897 | 0.037041 | 0.192461 | KNN |
| Floral diameter | 0.28302 | 0.034044 | 0.18451 | GBT |
| Floral diameter | 0.294681 | 0.03349 | 0.183004 | KRR |
| Relative fresh weight | 0.261311 | 0.786578 | 0.886892 | RF |
| Relative fresh weight | 0.140022 | 0.91573 | 0.956938 | XG |
| Relative fresh weight | 0.105028 | 0.952992 | 0.976213 | LR |
| Relative fresh weight | 0.002503 | 1.062163 | 1.030613 | PR_2 |
| Relative fresh weight | 0.002503 | 1.062163 | 1.030613 | PR_3 |
| Relative fresh weight | 0.036095 | 1.026394 | 1.013111 | Ridge |
| Relative fresh weight | 0.417253 | 0.620526 | 0.787735 | ANN |
| Relative fresh weight | 0.385013 | 0.654856 | 0.809232 | SVR |
| Relative fresh weight | 0.290723 | 0.755259 | 0.869056 | KNN |
| Relative fresh weight | 0.08929 | 0.969751 | 0.984759 | GBT |
| Relative fresh weight | 0.158554 | 0.895996 | 0.94657 | KRR |


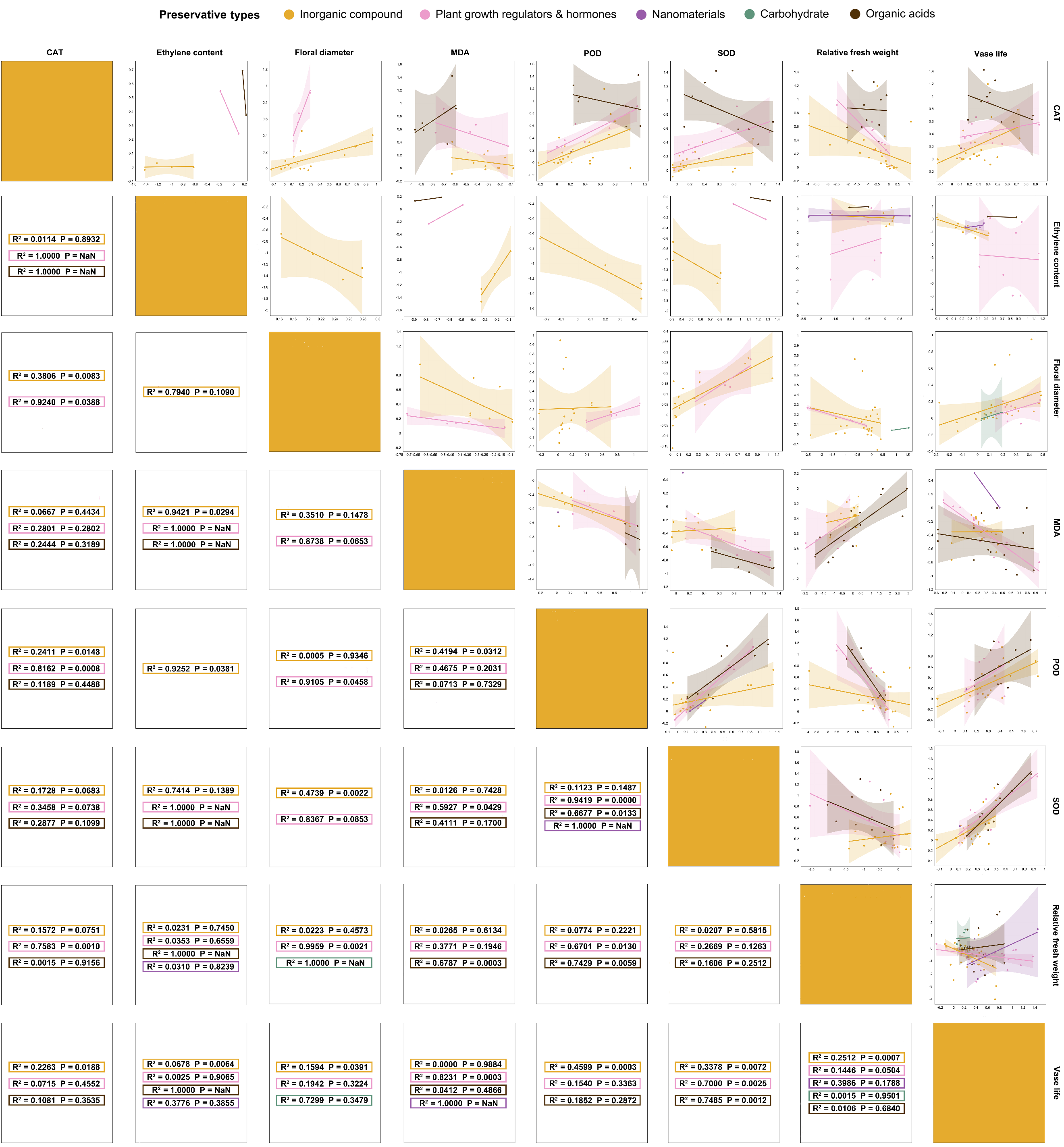


**Figure S1**. Linear relationship plot between the eight physiological parameters’ lnRR based on the preservative types. *P* < 0.05 indicates statistical significance. R^2^ indicates the fit goodness in the results of the linear fit.


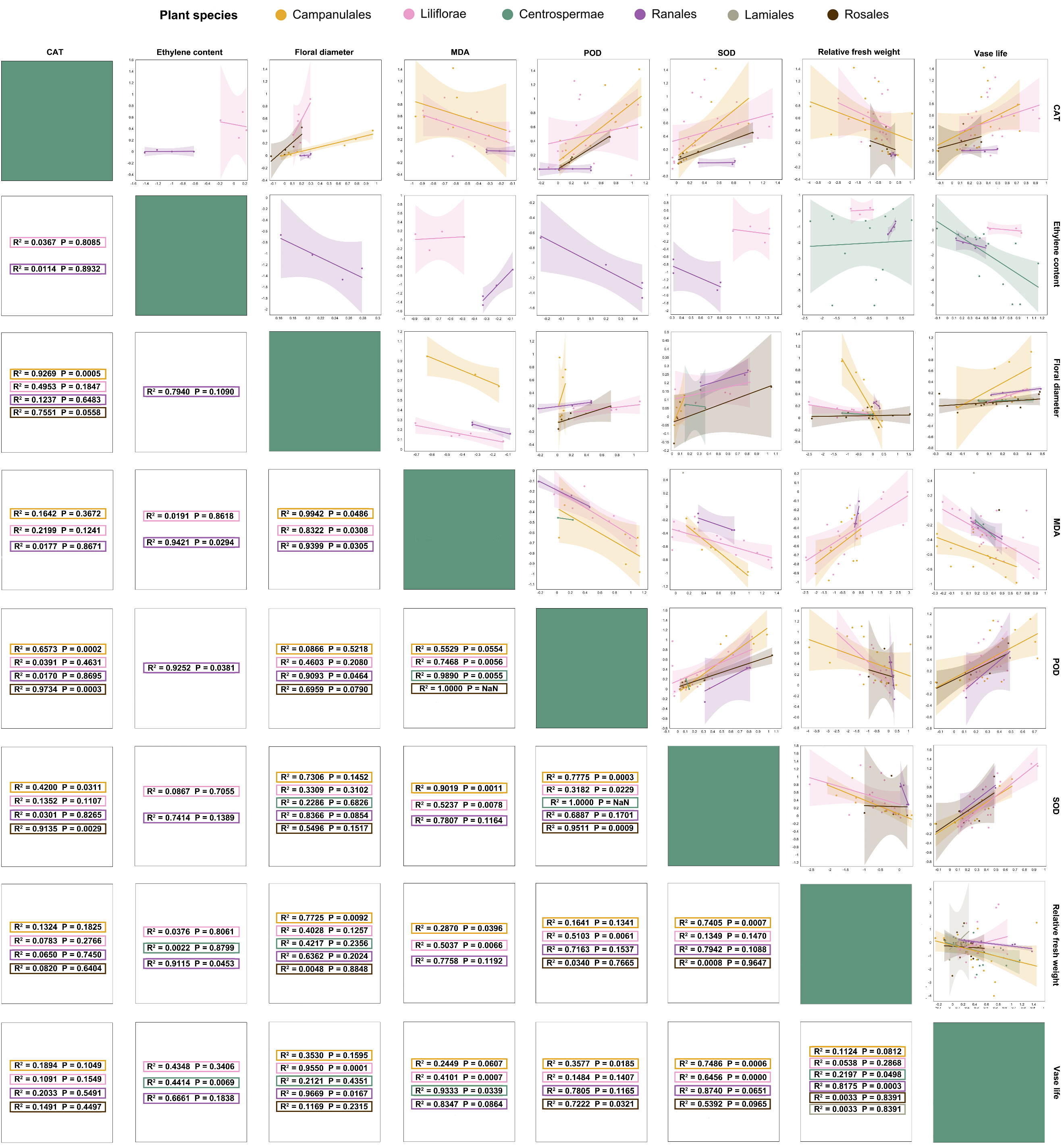


**Figure S2**. Linear relationship plot between the eight parameters’ lnRR based on the plant species. *P* < 0.05 indicates statistical significance. R^2^ indicates the fit goodness in the results of the linear fit.


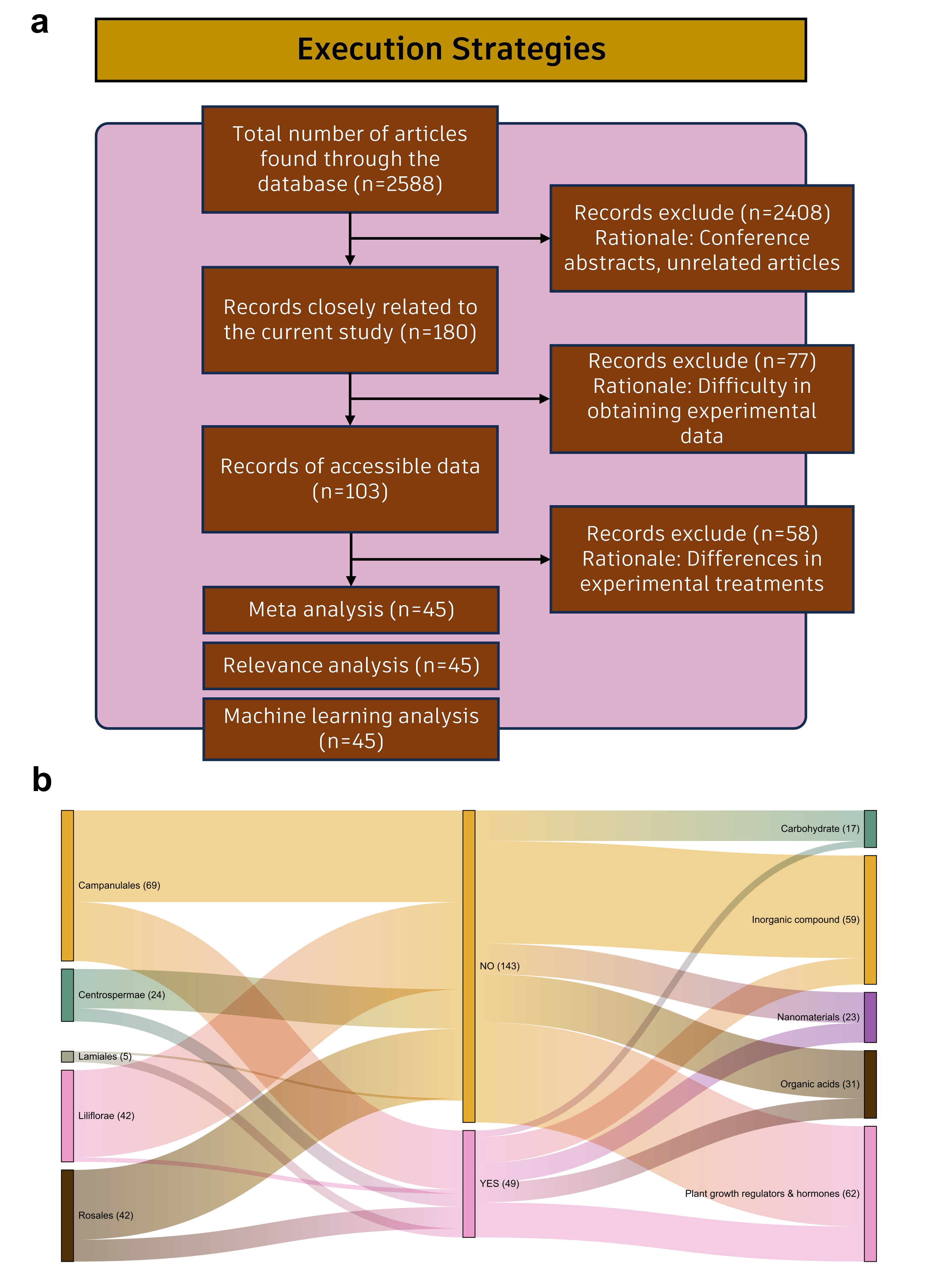


**Figure S3**. (a) Execution strategy for this investigation. (b) t-SNE visualization of data set interrelationships among plant species, pulse treatments, and preservative types.
